# Prevalence of HIV in a Prison of Tehran by Active Case Finding

**Published:** 2017-03

**Authors:** Seyed Ahmad SEYEDALINAGHI, Behnam FARHOUDI, Minoo MOHRAZ, Mona MOHAMMADI FIROUZEH, Mostafa HOSSEINI, Kianoush KAMALI

**Affiliations:** 1. Iranian Research Center for HIV/AIDS, Iranian Institute for Reduction of High-Risk Behaviors, Tehran University of Medical Sciences, Tehran, Iran; 2. Clinical Research Development Center, Amir-Almomenin Hospital, Tehran Medical Sciences Branch, Islamic Azad University, Tehran, Iran; 3. Dept. of Epidemiology and Biostatistics, School of Public Health, Tehran University of Medical Sciences, Tehran, Iran; 4. AIDS Office, Center for Communicable Diseases Control, Ministry of Health and Medical Education, Tehran, Iran

## Dear Editor-in-Chief

Prisoners worldwide, especially those with substance abuse, are at risk for acquiring HIV infection. Due to an insufficient supply of sterile needles and syringes in prisons, inmates are more inclined to share needles amongst themselves. A single needle is shared between 15 or 20 inmates. Some prisoners resort to making their own syringes with hardened plastic, resulting in damage to veins (1, 2).

There is a correlation between homelessness and HIV among prisoners. Certain behavioral and demographic characteristics are related to HIV infection among the homeless, including low education level, substance abuse, and unprotected sex. Such high-risk behaviors and limited access to health care services and condoms increase their risk of HIV transmission (3).

In Iran, prevalence of HIV infection is low among the general population and concentrated among injection drug users (IDUs) (4). Therefore, in order to prevent future HIV infections, both the general population and IDUs be educated on the risks of needle sharing. The establishment of treatment and prevention programs in prisons can also serve to limit HIV infection. Upon admission into prison, HIV screening can help detect undiagnosed prisoners. This will allow newly admitted prisoners to initiate antiretroviral therapy before the onset of fatal opportunistic infections. Controlling the virus at early stages can lead to a lower viral load and ultimately prevent HIV from spreading as effectively (5).

We aimed to assess the prevalence of HIV on male prisoners in the Great Tehran Prison from Oct 2013 to May 2014 using the active case finding (ACF) strategy.

The institutional review board of Tehran University of Medical Sciences approved the study. Active case finding of HIV was employed in the units by the prison’s clinical staff who educated the prisoners about HIV-related high-risk behaviors. All prisoners with risky behaviors were referred to the prison triangular clinic.

Table 1 shows the frequency of HIV infection by rapid test in three units of the prison. Briefly, HIV prevalence in units of one, two and three of the prison based on the ACF were 1.2%, 1.3%, and 1.9%, respectively.

**Table 1:** Description of active case findings for HIV in the units of the Great Tehran Prison

**Unit of the prison**	**Active case finding (n)**	**Released persons n (%)**	**People who declined test n (%)**	**All HIV^+^ patients n (%)**
Unit 1	496	4 (0.8)	2 (0.4)	6 (1.2)
Unit 2	550	12 (2.2)	3 (0.6)	7 (1.3)
Unit 3 (bands) *	693	53 (7.7)	2 (0.3)	12 (1.9)
Unit 3 (admitted) *	2050	355 (17.3)	149 (7.3)	(3.2) 35

* The first one is among who were present in the band and the second one (admission part) is among newly admitted prisoners.

Of the estimated 10.2 million prisoners worldwide, found that 3.8% are HIV positive (6). In Estonia, up to 90% of inmates were HIV positive in 2004 (7). In some countries, HIV infection has been controlled among prison communities. For instances, HIV prevalence among inmates in Australia is near zero (6).

Two huge outbreaks of HIV were reported in Iranian prisons. These outbreaks led to the development of new rules and policies to allow the introduction of needle exchange programs and widespread scale up of methadone treatment in prisons (8). HIV prevalence during admission time and annual rate of HIV were 24.4% and 16.8% (9).

Prisons are a good environment to introduce ACF in order to detect HIV infection among prison admission in order to achieve higher coverage of HIV care and treatment services.
